# Computed Tomography-Guided Methylene Blue Localization: Single vs. Multiple Lung Nodules

**DOI:** 10.3389/fmed.2021.661956

**Published:** 2021-04-14

**Authors:** Chia-Ying Lin, Chao-Chun Chang, Li-Ting Huang, Ta-Jung Chung, Yi-Sheng Liu, Yi-Ting Yen, Yau-Lin Tseng

**Affiliations:** ^1^Department of Medical Imaging, College of Medical College, National Cheng Kung University Hospital, National Cheng Kung University, Tainan, Taiwan; ^2^Division of Thoracic Surgery, Department of Surgery, College of Medical College, National Cheng Kung University Hospital, National Cheng Kung University, Tainan, Taiwan; ^3^Division of Trauma and Acute Care Surgery, Department of Surgery, College of Medical College, National Cheng Kung University Hospital, National Cheng Kung University, Tainan, Taiwan

**Keywords:** dye, localization, computed tomography, pulmonary nodule, lung cancer surgery

## Abstract

**Background:** Preoperative localization for small invisible and impalpable pulmonary nodules is important in single-port video-assisted thoracoscopic surgery (VATS). Localization of multiple pulmonary nodules during VATS resection remains challenging. The aim of our study is to elucidate the efficacy of preoperative CT-guided methylene blue localization of both single and multiple pulmonary nodules.

**Methods:** Consecutive patients undergoing preoperative CT-guided methylene blue dye localization for lung nodules, followed by VATS resection, were retrospectively analyzed between January 2014 and November 2019. Chi-square tests, Fisher's exact test and independent *T-*test were used to compare variables between the groups. Logistic regression was used to identify risk factors for procedure-related complications.

**Results:** A total of 388 patients, including 337 with single nodule and 51 with multiple nodules, were analyzed. The success rate of preoperative CT-guided methylene blue localization for both single and multiple pulmonary nodules were comparable as 98.8% (333/337) vs. 100% (108/108). The procedure time was longer (23.2 ± 9.4 vs. 7.6 ± 4.8 min, *p* < 0.001) and risk of pneumothorax was higher (47.1 vs. 25.5%, *p* = 0.002) in the multiple nodule group. The procedure time (OR 1.079; 95% CI = 1.041–1.118; *p* < 0.001) was an independent risk factor for pneumothorax. Nodule depth (OR 2.829; 95% CI = 1.259–6.356; *p* = 0.011) was an independent risk factor for pulmonary hemorrhage.

**Conclusions:** Preoperative CT-guided methylene blue localization for both single and multiple pulmonary nodules is safe, feasible, and effective.

## Introduction

Lung cancer is the leading cause of cancer-related death worldwide (1). With the increase of low-dose CT screening for lung cancer, small and multiple pulmonary nodules are frequently detected (2). There is also growing population of patients diagnosed as having multiple primary lung cancer. The limited surgical resection not only provides early diagnosis but is a potential curative method for these small lesions (3). Recent studies suggest that surgery for patients with multiple primary lung cancer is associated with relatively good 5-year survival outcomes (4). In patients with certain malignancies such as colorectal, renal cell carcinoma, melanoma, soft tissue sarcoma, head and neck squamous cell carcinoma, breast cancer and heptotocellular carcinoma, pulmonary metastectomy provides long-term survival benefit when there is good control of the primary cancer and extrathoracic metastasis (5–8). The transthoracic needle biopsy, however, has a limited yield rate for small and multiple nodules (9). The precise preoperative localization procedures for impalpable small lesions are therefore mandatory to achieve adequate resection and to avoid unnecessary pulmonary resection.

Accurate localization of small nodules using VATS has been reported to be challenging, especially for multiple, deep-seated or ground-glass nodules (10, 11). The methylene blue dye localization of pulmonary nodules was first reported in 1994 (12), with high success rate and short procedure time via both transthoracic and endobronchial route under CT or fluoroscopic guidance (13, 14). Although preoperative dye localization for lung nodules has been previously studied (10, 11, 13, 15–19), there was limited research focusing on the efficacy and feasibility (20). In the past, various CT-guided localization methods existed among different interventional radiologists in our hospital, resulting in unsteady, and inconsistent yield rate. An algorithmic approach was therefore proposed to standardize the localization procedure and to improve the outcomes. The aim of this study is to investigate the safety, feasibility, and efficacy of preoperative CT-guided methylene blue localization for both simple and multiple pulmonary nodules using an algorithmic approach.

## Methods

### Study Population

This retrospective study was approved by the institutional review board (A-ER-106-325) and informed consent was waived. Between January 2014 and November 2019, patients with undiagnosed pulmonary nodules undergoing preoperative CT-guided methylene blue dye localization followed by single-port VATS wedge resection at National Cheng Kung University Hospital were analyzed.

The surgical indications of small pulmonary nodules included (a) persistence of a nodule with interval growth, (b) development of solid component in a nodule with ground-glass opacity, (c) nodules suspected of pulmonary metastasis, and (d) a small extralobar lung lesion in patients harboring lung cancer deemed for lobectomy (wedge resection concomitantly with lobectomy) in order to confirm the diagnosis [i.e. metastatic lesion, multiple primary lung cancer [MPLC] or other benign entity]. The indication for preoperative CT-guided dye localization includes nodules with ground-glass opacity or maximal diameter < 10 mm.

The age, sex, smoking history, number and characteristics of the nodules, presence of perilesional emphysema, preoperative tentative diagnosis, patients' position, procedure time, time to operation, operative time for the total surgical procedure and wedge resection and procedure-related complications such as pneumothorax and pulmonary hemorrhage were recorded. The procedure time was defined as the time interval between the injection of local anesthesia and the final set of CT images. The time to operation was defined as the time interval between the final set of CT imaging and the first skin incision. The single nodule group was defined as patients with single nodule that required localization. The multiple nodule group was defined as patients with multiple nodules that required localization.

### CT-Guided Methylene Blue Dye Localization

Preoperative CT-guided localization was performed by one of four board-certified radiologists (T.J.C., H.L.K., C.Y.L. and L.T.H., with 19, 11, 5, and 5 years of experience, respectively). The localization scans were done without contrast enhancement on a 64-slice CT scanner (Optima 660, General Electric, Milwaukee, WI, USA) with slice thickness of either 2.5 or 5 mm. Before CT-guided localization procedure, the patients were placed in supine, prone, or lateral decubitus position based on the planned needle trajectory.

**Figure 1:**
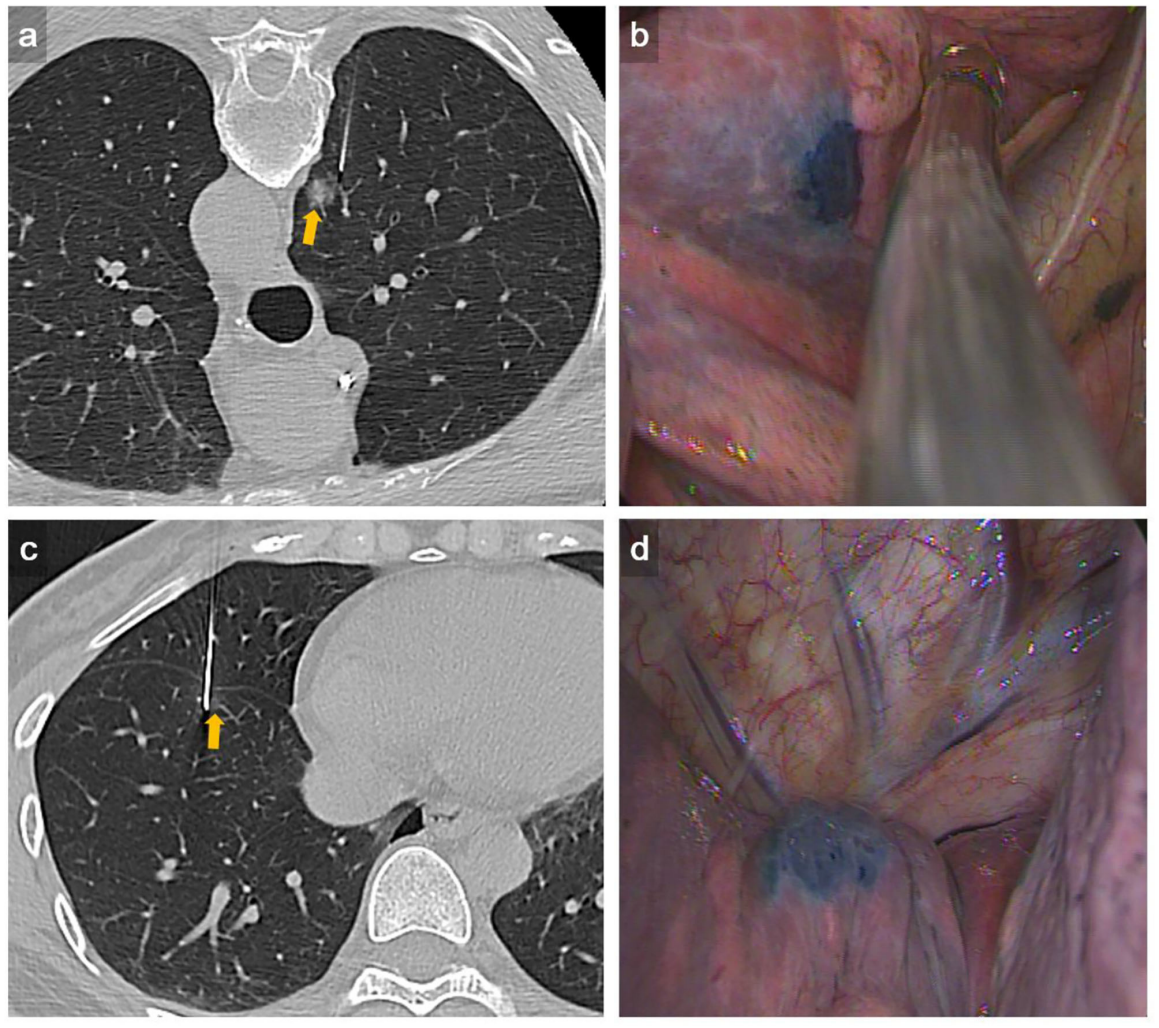
Representative example of complex dye localization. **(a)** An axial CT imaging with lung window during localization showed a 22-gauge Chiba needle introduced tangentially to the RUL part-solid lung nodule (arrow). **(b)** VATS imaging showed deposition of methylene blue on the RUL lung surface seen on the visceral pleura. The pathology result revealed adenocarcinoma. **(c)** An axial CT imaging with lung window during localization showed a 22-gauge Chiba needle introduced to the RLL GGN using trasfissural approach (arrow). Using this method, a total of three times of pleural puncture was done. **(d)** VATS imaging showed deposition of methylene blue on the RLL lung surface seen on the visceral pleura adjacent to right major fissure. The pathology result revealed AAH.

Simple site of localization was defined as nodules located in the outer surface of pulmonary lobe adjacent to the costal pleura (i.e., < 2 cm from costal pleura) and were approached by direct perpendicular route. Complex site of localization was defined as nodules located in the inner surface of pulmonary lobe adjacent to mediastinal pleura, diaphragmatic pleura, or the fissure. In these locations, there was no available direct perpendicular route, and the nodules were approached by tangential route or transfissural approach (Figure 1). When performing complex site localization, radiologist would communicate with surgeon about the relationship of tagged pleural surface to the nodule. Afterwards, surgeon could understand the direction in which wedge should be done relative to the methylene blue tract.

**Figure 2:**
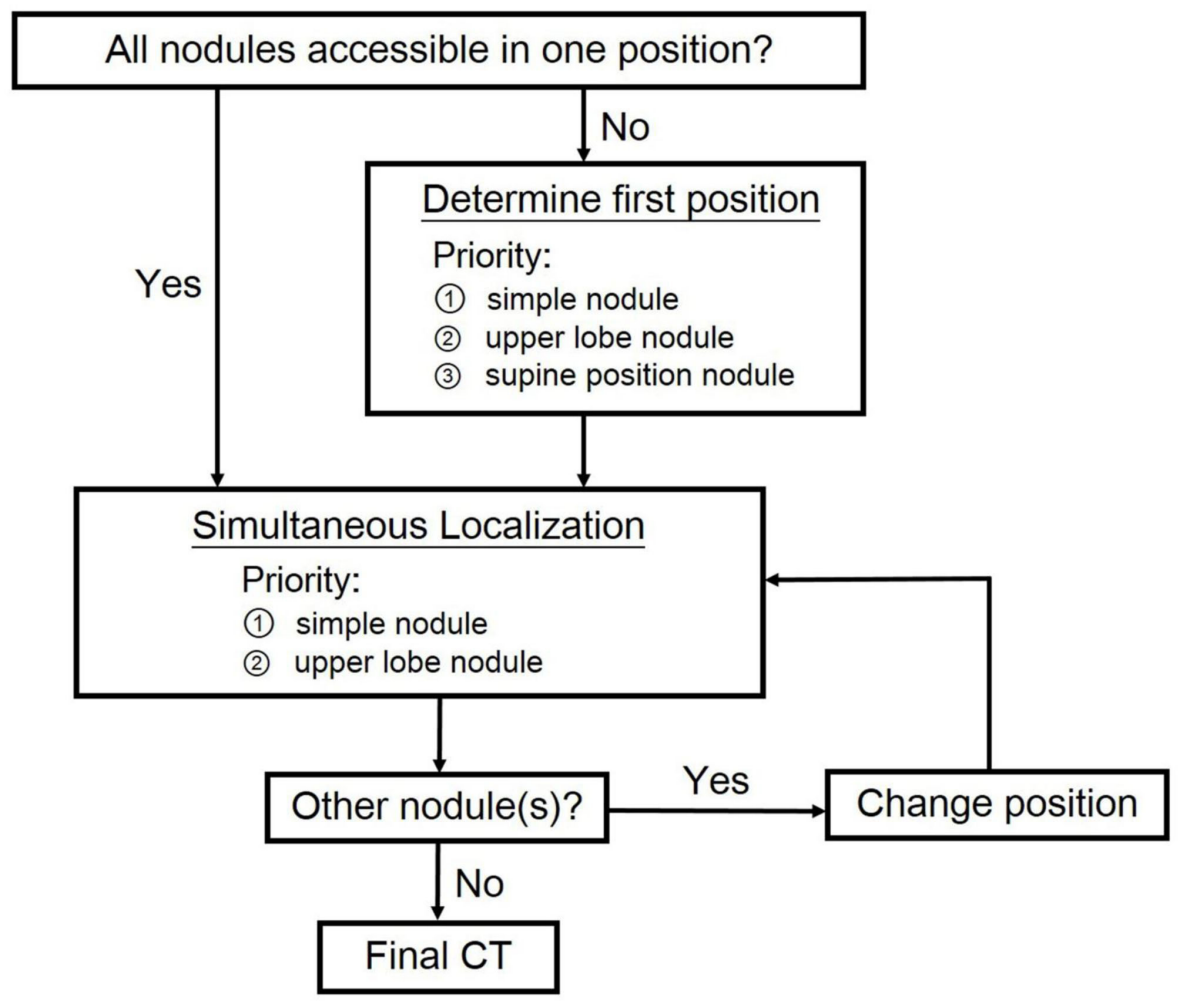
Algorithm proposed for dye localization of multiple pulmonary nodules.

For patients undergoing multiple localization, our algorithm was shown in Figure 2. The initial position for procedure was determined to include as many nodules at simple sites as possible. Simple site localization would become difficult once pneumothorax happened. On the other hand, complex site localization was done via long tract in aerated lung parenchyma and therefore adjustment of needle was possible when pneumothorax occurred. If simultaneous localization for multiple nodules was possible, nodules at simple sites in the upper lobes were to be localized first because of the relatively small lung volume variation on respiration. Multiple needles were used in case of multiple localization. All of them were left in place, not being withdrawn and reinserted. Needles were removed simultaneously after all localization was completed to avoid early pneumothorax. If simultaneous localization was not possible, our priority is to puncture simple site or upper lobe lesion at the first position. Once the localization was completed, the needle was removed and the patient repositioned for localization of the rest of nodules.

After patient positioning, under local anesthesia and aseptic condition, a 21- or 22-gauge Chiba needle was inserted into the lung nodule. As soon as the location of the needle tip had been confirmed, 0.3–0.5 ml of sterile methylene blue dye (10 mg/mL) was injected into the nodule. After the localization procedure had been completed, a final set of CT scan was done to detect potential complications. Afterwards, the patient was transferred to the operating room.

### VATS After Localization

**Figure 3:**
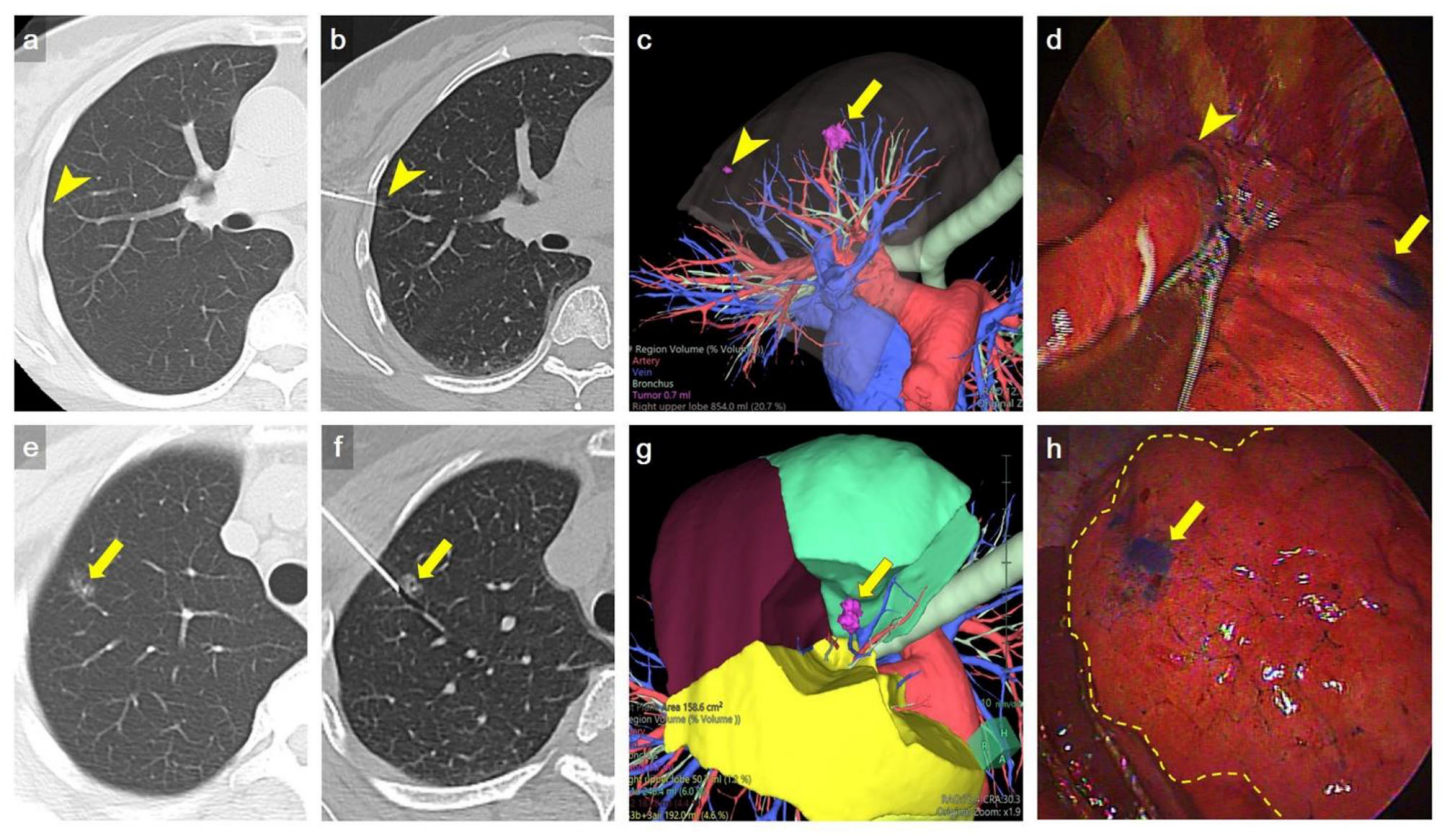
Representative example of multiple nodule dye localization. **(a,e)** Axial CT imaging with lung window showed two ground-glass nodules in RUL (arrowhead and arrow, respectively). **(b,f)** An axial CT imaging with lung window during localization showed 22-gauge Chiba needles introduced to the RUL nodules, respectively (arrowhead and arrow). **(c,g)** Preoperative 3D volume rendering (VR) imaging showed the location of RUL nodules (arrow and arrowhead). **(d,h)** VATS imaging showed deposition of methylene blue on RUL (arrow and arrowhead). The inflation-deflation intersegmental plane was shown as dash line in h. Wedge resection of a tiny RUL GGN (arrowhead) was done first, followed by RS1b+3ai subsegmentectomy for a larger RUL GGN (arrow) to ensure adequate deep margin. The location of dye marking correlated well with preoperative 3D VR imaging. The pathology result revealed AIS and MIA, respectively.

Patients were transferred to the operating room after CT-guided dye localization, followed by generalized anesthesia with selective lung ventilation. VATS were all performed on the same day immediately after localization. The pulmonary lesion was identified by visualization of the methylene blue dye on the lung surface. The depth of resection was determined based on pre-procedure CT scan. After identification of the marked nodule by thoracoscopic vision, wedge resection was performed using an endoscopic stapler with estimated sufficient resection margin from the lesion. Single-port VATS wedge resection was attempted for all the localized nodules. For nodules not well-localized for wedge resection or with suspiciously inadequate margin, sublobar anatomic resection such as segmentectomy or subsegmentectomy was performed instead (Figure 3).

### Success Rate and Complication

Technical failure was defined as (a) inability of intraoperative recognition of precisely dye-labeled area, (b) inadequate surgical margins in pathology, or (c) no nodule in the wedge resection specimen. The grade of pneumothorax was graded according to the 2010 British Thoracic Society guideline (21), the differentiation of a “large” from a “small” pneumothorax was defined as presence of a visible rim of > 2 cm between the lung margin and the chest wall at the hilum level.

The severity of pulmonary hemorrhage was graded according to a grading scheme modified by Tai et al. (22). Grade 0 was defined as no pulmonary hemorrhage, grade 1 as needle tract hemorrhage 2 cm or less in width, grade 2 as hemorrhage more than 2 cm in width but sublobar, grade 3 as lobar hemorrhage or greater, and grade 4 as hemothorax. The sex, age, smoking history, procedure time, multiple localization, number of pleural puncture, position, nodule location, nodule size, nodule depth, complex site, and lesion characteristics were included in the risk analysis for pneumothorax and pulmonary hemorrhage.

### Statistical Analysis

Patient demographics and nodule characteristics were summarized using descriptive statistics (mean ± SD for continuous variables, and proportions for categorical variables). Chi-square tests, Fisher's exact test, and independent *T-*test were conducted, respectively, to evaluate the discrete and continuous variables of patient's characteristics. Logistic regression was used to identify risk factors for pneumothorax and pulmonary hemorrhage. A *p* ≤ 0.05 was set to indicate statistical significance. SPSS system (IBM SPSS Statistics, Version 22.0, Armonk, NY) was used for statistical analysis.

## Results

### Patient and Lesion Characteristics

Demographic characteristics were detailed in Table 1. A total of 388 consecutive patients were eventually analyzed. There were 337 patients in the single nodule group, and 51 in the multiple nodule group. In the multiple nodule group, 47 patients had 2 nodules, 3 patients had 3 nodules, and 1 patient had 5 nodules. There was no significant difference in age, gender, smoking history, time to operation between the two groups. None of the patients had perilesional emphysema. Sixteen patients in the multiple nodule group (31.4%) needed to change their position during the dye localization procedure. The procedure time was significantly longer in the multiple nodule group than that in the single nodule group (23.2 ± 9.4 vs. 7.6 ± 4.8 min, *p* < 0.001). The time to operation was not different between the two groups (163.2 ± 83.2 min in the single nodule group and 164.6 ± 83.8 min in the multiple nodule group, *p* = 0.913). The preoperative tentative diagnosis included primary lung neoplasm, metastasis from other origin, and infection or inflammation. In the single nodule group, the tentative diagnosis was primary lung neoplasm in 28 (67.7%) patients, metastasis in 41 (12.2%) patients, and infection or inflammation in 68 (20.2%) patients. In the multiple nodule group, the tentative diagnosis was primary lung neoplasm in 40 (78.4%) patients, metastasis in 6 (11.8%) patients, and infection or inflammation in 5 (9.8%) patients. There was no significant difference in preoperative tentative diagnosis between two groups. There was no significant difference in operative time (*p* = 0.772) between single nodule group (92.2 ± 65.2 min) and multiple nodule group (88.8 ± 57.7 min). Excluding patients undergoing procedures other than pulmonary resection (e.g., anatomic resection and thymothymectomy), the operative time was comparable (*p* = 0.770) between single nodule group (63.6 ± 30.0 min, *n* = 275), and multiple nodule group (65.3 ± 36.0 min, *n* = 34).

**Table 1:** Demographics of patients undergoing CT-guided dye localization for surgery.

**Variables**	**Single**	**Multiple**	***p***
	**(*n =* 337)**	**(*n =* 51)**	
Age (years)	58.6 ± 11.0	57.6 ± 10.4	0.542
Gender (male, %)	105 (31.2%)	19 (37.3%)	0.478
Smoking	87 (25.8%)	16 (31.4%)	0.399
**Number of patients with**			
2 nodules		47	
3 nodules		3	
5 nodules		1	
**Position**			
Supine	221 (65.6%)	37 (72.5%)	0.410
Prone	116 (34.4%)	29 (56.9%)	0.003
Decubitus	0 (0%)	1 (2.0%)	0.131
Reposition during procedure		16 (31.4%)	
**Preoperative tentative diagnosis**			0.476
Primary lung neoplasm	228 (67.7%)	40 (78.4%)	
Metastasis	41 (12.2%)	6 (11.8%)	
Infection/ inflammation	68 (20.2%)	5 (9.8%)	
Procedure time (min)	7.6 ± 4.8	23.2 ± 9.4	<0.001
Time to operation (min)	163.2 ± 83.2	164.6 ± 83.8	0.913
Number of pleural puncture	1.02 ± 0.20	2.24 ± 0.71	<0.001
Pneumothorax	86 (25.5%)	24 (47.1%)	0.002
Operative time (min)
Total^*^	92.2 ± 65.2	88.8 ±57.7	0.722
Wedge resection^**^	63.6 ± 30.0	65.3 ± 36.0	0.770

*Other primary lung neoplasm including carcinoid tumor, squamous cell carcinoma, lymphoepithelioma-like carcinoma*.

* *total included anatomic resection*.

** *wedge resection excluded patients underwent anatomic resection and thymothymectomy (n = 275 in single nodule group, n = 34 in multiple nodule group)*.

Lesion characteristics were detailed in Table 2. There was no significant difference in lung nodule size, depth, location (including complex site), lesion characteristics, pathology results, or margin between the two groups. The mean tumor size was 5.7 ± 2.9 mm in the single nodule group and 5.2 ± 3.4 mm in the multiple nodule group. The nodule depth measured from visceral pleura was 6.1 ± 5.4 mm in the single nodule group and 5.6 ± 5.9 mm in the multiple nodule group. The majority of nodules presented on the CT scan with ground glass appearance, with 60.2% in the single nodule group and 58.3% in the multiple nodule group. Pathologic diagnosis revealed that adenocarcinoma *in situ* (AIS), atypical adenomatous hyperplasia (AAH) and benign lesions (including anthracosis, pulmonary meningothelial-like nodule, chronic inflammation, fibrosis, sclerosing pneumocytoma, bronchial metaplasia, bronchial adenoma, hamartoma) were common in both groups (18.7, 16.9, and 27.6% in single nodule group vs. 23.4, 22.4, and 20.6% in multiple nodules group). The resection margin was comparable between two groups, 0.77 ± 0.67 cm in single nodule group and 0.96 ± 0.75 cm in multiple nodules group (*p* = 0.081).

**Table 2:** Characteristics of pulmonary nodules and results of CT-guided dye localization.

**Variables**	**Single**	**Multiple**	***p***
	**(*n =* 337)**	**(*n =* 108)**	
Size (mm)	5.7 ± 2.9	5.2 ± 3.4	0.151
Depth (mm)	6.1 ± 5.4	5.6 ± 5.9	0.367
**Location**			0.928
RUL	104 (30.9%)	30 (27.8%)	
RML	29 (8.6%)	11 (10.2%)	
RLL	68 (20.2%)	25 (23.1%)	
LUL	75 (22.3%)	23 (21.3%)	
LLL	61 (18.1%)	19 (17.6%)	
**Lesion characteristic**			0.180
Ground glass	203 (60.2%)	63 (58.3%)	
Part solid	68 (20.2%)	31 (28.7%)	
Solid	65 (19.3%)	14 (13.0%)	
Cavitation	1 (0.3%)	0 (0%)	
**Pathology**			0.718
Adenocarcinoma	31 (9.2%)	9 (8.3%)	
MIA	52 (15.4%)	16 (14.8%)	
AIS	63 (18.7%)	25 (23.1%)	
AAH	57 (16.9%)	25 (23.1%)	
Metastasis	27 (8.0%)	7 (6.5%)	
Granuloma	8 (2.4%)	3 (2.8%)	
Benign lesions	93 (27.6%)	22 (20.4%)	
Other primary lung neoplasm	6 (1.8%)	1 (0.9%)	
Resection margin (cm)	0.77 ± 0.67	0.96 ± 0.75	0.081
Pulmonary hemorrhage	13 (3.9%)	1 (0.9%)	0.204
Complex site	32 (9.5%)	16 (14.8%)	0.170
Success	333 (98.8%)	108 (100%)	0.576

### Success Rate

The rates of successful localization, confirmed intraoperatively, were 98.8% (333/337) in the single nodule group and 100% (108/108) in the multiple nodules group (Table 2). There was adequate resection margin in both groups (0.77 ± 0.67 cm in the single nodule group, 0.96 ± 0.75 cm in the multiple nodules group, *p* = 0.081). Four patients had failed dye localization. One patient had extensive anthracosis on the visceral pleura, making the localization indistinguishable. Another patient had failed localization due to rapid diffusion of methylene blue. Another patient had dye spillage on the parietal pleura. The other one had failed localization because of misplaced trajectory (RUL instead of superior segment of RLL due to needle tip traversing fissure). For these four patients with failed dye localization, the surgery was transformed from wedge resection to sublobar anatomic resection as segmentectomy or subsegmentectomy. None of the single-port VATS resection converted to multiple-port VATS resection because of localization failure.

### Complications

Pneumothorax is the major complication of CT-guided dye localization. The risk of pneumothorax was higher in the multiple nodule group (47.1 vs. 25.5%, *p* = 0.002) (Table 1). The majority of these patients had small pneumothorax and were asymptomatic, without the need of oxygen supplement or different anesthetic induction method. Only one patient in the single nodule group had large pneumothorax and received pre-operative pigtail insertion. Thirteen patients in the single nodule group and one patient in the multiple nodule group had procedure-related pulmonary hemorrhage identified on the final set of CT scan, and all of them were asymptomatic.

Logistic regression was used to identify risk factors for pneumothorax and pulmonary hemorrhage. For pneumothorax, univariate analysis revealed sex (male/female) [Odds ratio [OR] = 0.633, *p* = *0*.032, 95% confidence interval [CI] = 0.417–0.962], procedure time (OR = 1.075, *p* < *0*.001, 95% CI = 1.051–1.100), multiple localizations (OR = 2.721, *p* ≤ 0.001, 95% CI = 1.735–4.267), and number of pleural puncture (OR = 2.025, *p* < 0.001, 95% CI = 1.497–2.740) significantly increased the risk of pneumothorax. In multivariate analysis, only procedure time (OR = 1.079, *p* < 0.001, 95% CI = 1.041–1.118) independently increased the risk of pneumothorax (Table 3).

**Table 3:** Risk factors for procedure-related pneumothorax.

**Variables**	**Univariate**		**Multivariate**	
	***p***	**OR (95% CI)**	***p***	**OR (95% CI)**
Sex (Ref: female)	0.032	0.633 (0.417–0.962)	0.418	0.825 (0.517–1.314)
Age (years)	0.113	1.015 (0.996–1.035)		
Smoking	0.070	1.503 (0.967–2.336)		
Procedure time (min)	<0.001	1.075 (1.051–1.100)	<0.001	1.079 (1.041–1.118)
Multiple localization	<0.001	2.721 (1.735–4.267)	0.464	0.712 (0.287–1.768)
Number of pleural puncture	<0.001	2.025 (1.497–2.740)	0.733	1.108 (0.616–1.992)
**Position**				
Supine	0.529	0.873 (0.572–1.332)		
Prone	0.640	1.102 (0.732–1.660)		
Decubitus	0.026	11.541 (1.335–99.742)		
**Location (Ref: RUL)**				
RML	0.913	2.403 (0.349–4.281)		
RLL	0.232	0.689 (0.374–1.270)		
LUL	0.912	1.033 (0.576–1.854)		
LLL	0.614	1.216 (0.568–2.603)		
Nodule size (mm)	0.403	0.746 (0.376–1.483)		
Nodule depth (mm)	0.288	1.211 (0.850–1.725)		
Complex site	0.534	0.808 (0.413–1.581)		
**Lesion characteristic (Ref: ground glass)**				
Partial solid	0.647	1.122 (0.686–1.836)		
Solid	0.771	0.922 (0.531–1.599)		
Cavitation	1.000	–		

For pulmonary hemorrhage, most of the patients had grade 1 pulmonary hemorrhage, except one patient had grade 2 pulmonary hemorrhage (i.e., none of the patients had grade 3 or grade 4 pulmonary hemorrhage). Nodule depth significantly increased the risk of pulmonary hemorrhage (OR = 2.829, *p* = 0.011, 95% CI = 1.259–6.356) (Table 4).

**Table 4:** Risk factors for procedure-related pulmonary hemorrhage.

**Variables**	**Univariate**		**Multivariate**	
	***p***	**OR (95% CI)**	***p***	**OR (95% CI)**
Gender (Ref: female)	0.144	0.325 (0.072–1.473)	0.098	0.257 (0.051–1.301)
Age (years)	0.537	1.016 (0.966–1.069)	0.428	1.023 (0.966–1.083)
Smoking	0.996	–		
Procedure time (min)	0.996	1.000 (0.945–1.058)		
Multiple localization	0.162	0.232 (0.030–1.796)		
Number of pleural puncture	0.948	1.024 (0.504–2.081)		
**Position**				
Supine	0.442	0.655 (0.223–1.924)		
Prone	0.811	1.140 (0.389–3.344)		
Decubitus	0.999	–		
**Location (Ref: RUL)**				
RML	0.072	5.351 (0.862–33.225)		
RLL	0.116	3.75 (0.712–19.759)		
LUL	0.755	0.680 (0.061–7.612)		
LLL	0.307	2.571 (0.420–15.730)		
Nodule size (mm)	0.171	0.185 (0.017–2.062)		
Nodule depth (mm)	0.087	1.940 (0.904–4.159)	0.011	2.829 (1.259–6.356)
Complex site	0.206	2.339 (0.629–8.700)	0.113	3.120 (0.768–12.674)
**Lesion characteristic (Ref: ground glass)**				
Partial solid	0.415	0.528 (0.114–2.452)		
Solid	0.615	0.665 (0.143–3.100)		
Cavitation	1.000	–		

## Discussion

Our results demonstrated that using an algorithmic approach, CT-guided methylene blue dye localization for multiple pulmonary lung nodules had similar success rate to single lung nodule, facilitating intraoperative VATS identification and resection. The complications were well-tolerated and managed with conservative treatment. Simultaneously detected by the CT scan, a large amount of pneumothorax could be promptly managed with CT-guided drainage tube insertion.

Performing VATS wedge resection for impalpable small nodule is challenging, especially during single port resection, where finger palpation is difficult (10, 11). Suzuki et al. reported a 63% failure rate to find nodules ≤ 10 mm and more than 5 mm deep in the lung parenchyma without localization in VATS (23). Accurate preoperative localization not only improves the efficiency of the surgery, but helps the pathologist identify the lesion in the resected specimen. In patients with lung cancer, accompanied small lung nodules pose clinical conundrum in lung cancer staging. These tiny nodules could be metastasis, benign lesion or another synchronous early stage primary lung cancer. Obtaining histopathological results of these small nodules was important in order to accurately stage the patient, select proper treatment regimens, predict survival and report comparable end results.

Various methods have been proposed to improve the accuracy of pre-operative lung nodule localization, including using dye, hooks, microcoils, radiopaque markers (e.g., lipiodol), Indocyanine green (ICG), or transbronchial dye injection (14). Hook-wire has been widely used for deeper lesions (24), but is not suitable for multiple nodule localization due to high risk of dislodgement [ranged from 2.4 to 7.5% (25, 26)], pulmonary hemorrhage and pneumothorax (13), especially when patient reposition is required during localization procedure. Although metallic microcoil allows multiple lesions to be localized, it is technically demanding (27), since intraoperative fluoroscopic guidance would be necessary, increasing radiation exposure to both the patient and the operator (28). In addition, microcoils are more expensive than methylene blue. Non-radiopaque marker such as ICG requires special near-infrared fluorescence (29), with limited tissue penetration at depths within 24 mm (30), which is not applicable in deeper nodule localization. Executing dye labeling via bronchoscopic approach takes more time than the percutaneous approach (31). Pneumothorax after needle puncture could be aggravated by positive airway pressure (32). Nonetheless, when intraoperative localization is to be performed in the hybrid room, localization after general anesthesia and double-lumen tube insertion was widely accepted (32–34). The use of hybrid room localization would potentially reduce not only the procedure time using general anesthesia for localization, but also the risk of pneumothorax by reducing the time to operation after pleural puncture. The imaging quality of cone beam CT in the operating room is not as good as multidetector CT, posing the risk of needle malposition and hampering precise localization for lesions at complex site. Moreover, the limited availability of hybrid operating room restricts the operating capability (13).

Preoperative methylene blue localization has several advantages. Multiple nodules localization could be done simultaneously as demonstrated in our algorithmic approach. The advantage of its simplicity by using CT equipment and Chiba needle reduces the radiation exposure for the operator during the procedure. No special equipment such as CT fluoroscopy, or hybrid operation room is required. The procedure time is shorter. Kleedehn et al. reported that methylene blue injection is as efficacious as hook-wire insertion but has less complications (17). Our technical success rate was 98.8% in single nodule group and 100% in multiple nodules group, which was comparable to prior study (11, 19). In our study, the success rate for complex site localization was 100%. Compared with prior studies (32, 33), the nodules were more superficial in our cohort, enabling high visibility of methylene blue and high success rate. In addition, conversion from intentional wedge resection to sublobar anatomic resection to ensure adequate deep margin also contributed to high success rate. With the improvement of VATS surgical techniques, almost all the thoracoscopic pulmonary resection including segmentectomy and subsegmentectomy could be accomplished using single-port VATS (35).

The disadvantages of dye localization are its rapid diffusion and poor visualization in severely anthracotic lung, which accounted for two failed dye localization in our study. Another drawback of methylene blue labeling is the inability to reveal the depth of the lesion beneath the visceral pleura surface, so the safety margin cannot be assessed before the histopathological examination. However, in our institution, sublobar anatomic resection such as segmentectomy or subsegmentectomy would have been otherwise performed instead of wedge resection if the nodule was located deeper than 2 cm from visceral pleura (35).

When performing multiple dye localization, after careful planning the needles trajectories, the operator could design a certain position in order to conduct two consecutive pleural punctures simultaneously, thereby minimizing the time lag between two punctures, and potentially decreasing the total number of CT scans through entire procedure and decreasing total procedure time. Under the circumstances where multiple nodules were to be labeled, meticulous planning for patient position and needle trajectories enables simultaneous puncture for different nodules and minimizes not only the total procedure time but also the number of CT scan for confirmation. In the patient who had five lung nodules, tattooed, lateral decubitus position was used, and five pleural punctures were carried out at same time of CT scan (Supplementary Figure 1). Theoretically, a lateral decubitus position allows multiple dye localization to be performed at the same CT scan, and can hence decrease procedure time and the risk of pneumothorax. Nevertheless, for clear and conscious patients in our practice, lateral decubitus position provides less comfortability and stability during the procedure. An air splint might ameliorate the discomfort and facilitate simultaneous localization on lateral decubitus position.

Our results showed that the nodule depth was associated with the risk of pulmonary hemorrhage. These procedure-related pulmonary hemorrhage were asymptomatic and managed conservatively. Older age and longer procedure time significantly increased the risk of pneumothorax. It is interesting that although multiple localization and number of pleural puncture were associated with pneumothorax in univariate analysis, in multivariate analysis, only the procedure time independently increased the risk of pneumothorax. This could be attributed to the association of multiple localization and number of pleural puncture with procedure time, whereas prolonged procedure time reflects the technical difficulty more than multiple localization and number of pleural puncture. Decreasing procedure time, therefore, might have played a vital role. Measures have been taken in our institute to decrease procedure time, including enhancing patients' education on consistency of breathing, utilization of needle guidance systems to improve needle tip positioning, reducing total number of needle passes and needle adjustments, and cohesive teamwork between radiologists, nurses and technicians to improve efficiency. Localization in a hybrid operating room under general anesthesia could also be the solution. Although the complications such as pneumothorax and hemorrhage are noteworthy, they do not impact the ability to perform the VATS wedge resection.

Our study had limitations. First, it was a retrospective and single center study with a relatively small population. There was no control group of VATS patients without preoperative localization, which may lead to overestimating the safety and effectiveness of preoperative algorithmic CT-guided methylene blue labeling for both single and multiple pulmonary nodules.

## Conclusion

In conclusion, although the procedure time and risk of pneumothorax is higher in multiple nodule group, preoperative algorithmic CT-guided methylene blue labeling for both single and multiple pulmonary nodules is considered safe and feasible, supporting successful VATS resection of small lung nodules.

## Data Availability Statement

The original contributions generated in the study are included in the article/Supplementary Material, further inquiries can be directed to the corresponding author.

## Ethics Statement

The studies involving human participants were reviewed and approved by National Cheng Kung University Hospital Institutional review board. Written informed consent for participation was not required for this study in accordance with the national legislation and the institutional requirements.

## Author Contributions

C-YL and C-CC were involved in data collection, study design, analysis, preparation, and review of manuscript. L-TH and T-JC were involved in data collection and review of manuscript. Y-LT, Y-SL, and Y-TY were involved in review of manuscript. All authors contributed to the article and approved the submitted version.

## Conflict of Interest

The authors declare that the research was conducted in the absence of any commercial or financial relationships that could be construed as a potential conflict of interest.

## Supplementary Material

Supplementary Figure 1 Performing dye localization for five left lung nodules using right decubitus position at the same CT scan. Five consecutive pleural punctures were done simultaneously. **(a–e)** Axial CT imaging with lung window during localization shows 22-gauge Chiba needles introduced adjacently to the targeted lung nodules in the LUL **(a,b)** and LLL **(c–e)**. The pathology results revealed AIS, AAH, MIA, MIA, and interstitial fibrosis, respectively.
