# Supplementary figures and images for: Computed Tomography-Guided Methylene Blue Localization: Single vs. Multiple Lung Nodules
Source: Front Med (Lausanne). 2021 Apr 14;8:661956. doi: 10.3389/fmed.2021.661956 (PMC8079624; doi:10.3389/fmed.2021.661956)

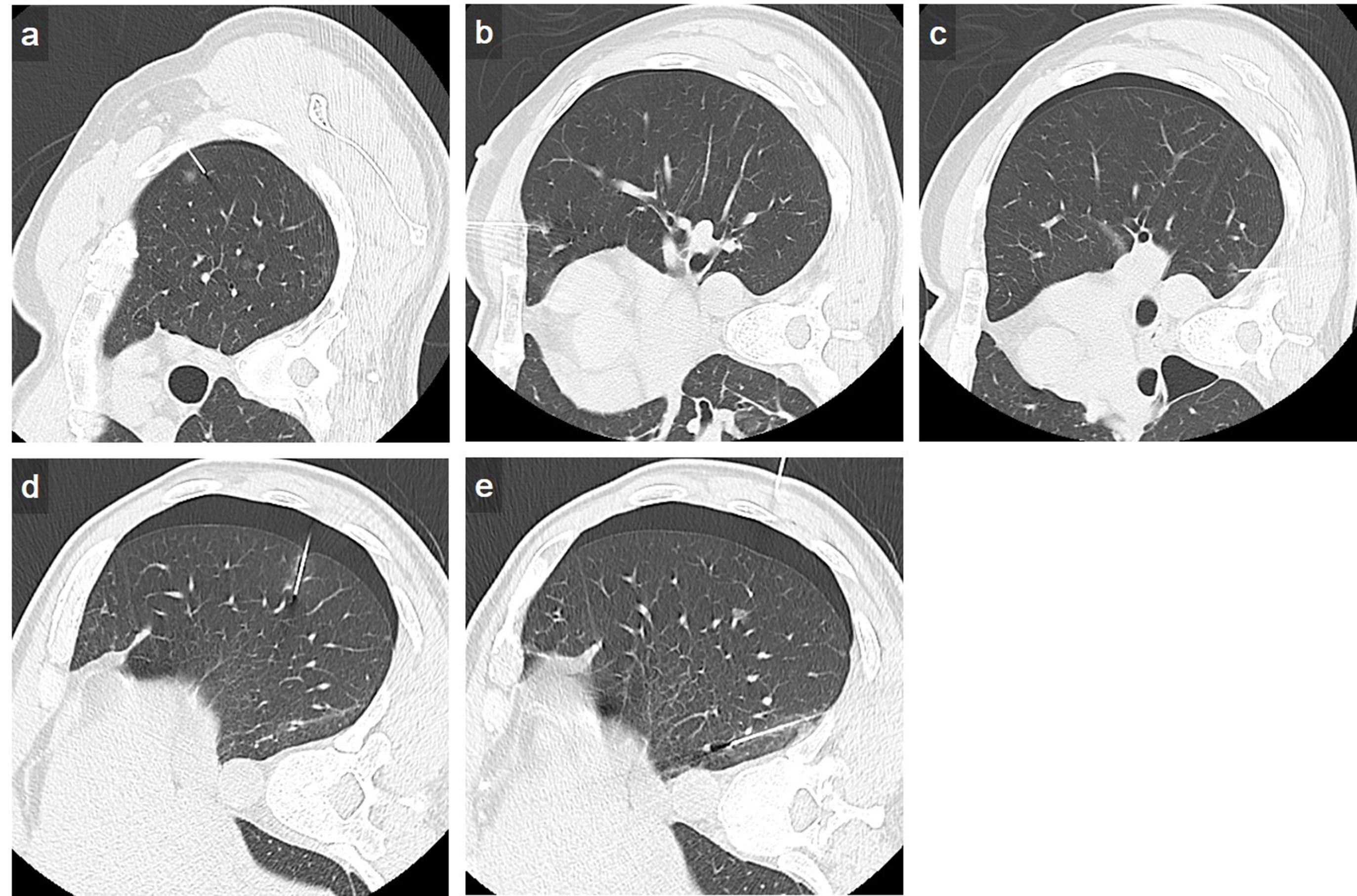

Supplement: Supplementary Figure 1 — Performing dye localization for five left lung nodules using right decubitus position at the same CT scan. Five consecutive pleural punctures were done simultaneously. (a–e) Axial CT imaging with lung window during localization shows 22-gauge Chiba needles introduced adjacently to the targeted lung nodules in the LUL (a,b) and LLL (c–e). The pathology results revealed AIS, AAH, MIA, MIA, and interstitial fibrosis, respectively. [file Image_1.JPEG]
